# The Institutional Treatment of Disease

**Published:** 1900-09-29

**Authors:** 


### The Institutional Treatment of Disease.

With the steady growth of specialism and the introduction of many new and often elaborate methods of treating disease, the question of establishing institutions in which these special methods may be carried out in a more complete manner than is possible in the ordinary homes of ordinary people becomes one of considerable importance. In every rank in life people are no longer content when ill to go to a general practitioner. No sooner do they find out what is the matter than off they go to some man whom they believe to be a "specialist" in their particular case. We do not grumble at them for so doing. It is one of the inevitable results of the modern tendency to division of labour which we see showing itself in every form of work. The difficulty lies in complying with the elaborate details of the prescriptions which patients nowadays receive. If they are poor they go to a hospital, and if they are rich they betake themselves to some possibly very expensive nursing home. But if they are neither poor nor rich they are in a quandary. They are told perhaps to have baths of a special kind, or inunctions of a peculiar description, which, if applied at home, would smell the house out, and maybe certain dressings are ordered which must be applied either by the medical practitioner himself or by some nurse specially skilled in their application. The prescription is useless without the means for carrying it out, and the day is coming when, far more than is the case at present, people will find themselves driven to go to institutions for medical treatment. We already have farm colonies for the treatment of epilepsy, sanatoria for the treatment of consumption, special bath places for the treatment of heart debility, and others for rheumatic and gouty conditions, special institutions for treatment by light, heat, oxygen, and electricity, and in every one of these the patient receives some special form of treatment which he would have found it difficult and probably impossible to obtain at home.

Of course, we are all accustomed to surgical homes, and the necessity of such establishments in surgical cases is generally admitted, but now the drift is towards the treatment of all sorts of other cases in special institutions, and the questions should be faced, Who is to run them? and Who is to be responsible for this institutional treatment which is coming into the market? There is no doubt that, notwithstanding the amount of money that might be made by running such establishments, the feeling of the medical profession in England is, as a whole, against medical men undertaking the work. But, then, the general feeling of the medical profession is always against innovations, and is never very kindly inclined towards specialism, of which the institutional treatment of disease is the latest outcome and the most advanced example. What, however, we have to recognise is, that special institutions can do better work than can be done by merely giving a man a prescription and leaving him to get it applied as best he can. Take the case of skin diseases. No doubt it is most "professional" for the physician to sit in his consulting-room and content himself with exchanging advice for guineas. Moreover, when the practice has once come it is the easier plan. But from the patient's point of view the German plan is by far the best. As Dr. Abraham has lately said, we are in this country handicapped in great degree in the treatment of diseases of the skin by the fact that we cannot have the various necessary measures properly carried out; the circumstances of the patient's home but rarely lend themselves to thorough inunction, suitable baths, &c., and in a large number of cases the result is that the case "hangs fire." The Continental patient suffering from a skin disease is, however, much better off. He also goes to an experienced special practitioner, but instead of contenting himself with giving him a prescription this gentleman admits him into his private hospital, at a cost varying according to his means. In this institution he is seen and dressed every day by the practitioner or by his assistants, with frequent modifications of treatment as indicated, "and I am obliged to admit," says Dr. Abraham, "is often cured more quickly and more satisfactorily than he would be in this country." The enterprising German, who certainly is quite as well up in professional matters as we are, when he has decided on a line of treatment does not think it beneath his dignity to carry it out, even though in so doing he may have to go down to the bath-house to see the bath administered, or may have to set up an institution of his own in which to carry out the special methods of cure which he has devised. In the eyes of the patients all this is counted unto him for righteousness, for they see in it a common-sense attempt to carry out the treatment in exactly the way intended. Moreover, his German colleagues do not look askance at the good man, knowing that there is cash in the process, and that they themselves would be only too glad to go and do likewise. To us, however, this mixture of divers profits, of fees, and lodgings, of treatment and board, all smells of quackery and commercialism. We will have none of it. So when our patients ask for treatment we turn them into the street with a prescription! Fashion rules in medicine just as it does in ladies' bonnets, and its dictates, whether issued in Paris or Pall Mall, are responsible for much. Just now the leading lights in medical "society," in whose footsteps we all so carefully tread, consider that trade does not "tone well" with medicine. Perhaps they are right. Perhaps, however, trade need not of necessity be so black as it is painted, and medicine may not always be so guileless as some pretend. We will not say. But in the meantime our patients go to Germany, and the institutional treatment of disease, which ought above all things to be carried out under the strictest medical control, is allowed in England to drift into the hands of outsiders.

